# SARS-CoV-2 Infection and Its Association with Maternal and Fetal Redox Status and Outcomes: A Prospective Clinical Study

**DOI:** 10.3390/jcm14051555

**Published:** 2025-02-26

**Authors:** Marija Bicanin Ilic, Tamara Nikolic Turnic, Igor Ilic, Aleksandar Nikolov, Srdjan Mujkovic, Dejana Rakic, Nikola Jovic, Neda Arsenijevic, Slobodanka Mitrovic, Marija Spasojevic, Jelena Savic, Katarina Mihajlovic, Nevena Jeremic, Jovana Joksimovic Jovic, Bozidar Pindovic, Goran Balovic, Aleksandra Dimitrijevic

**Affiliations:** 1 Faculty of Medical Sciences, Department of Gynecology and Obstetrics, University of Kragujevac, 34000 Kragujevac, Serbia; aleksandar.nikolov2@gmail.com (A.N.); drsrdjanmujkovic@gmail.com (S.M.); dejavulovic@gmail.com (D.R.); docctorny@gmail.com (N.J.); velickovicneda@gmail.com (N.A.); saskadkg@gmail.com (A.D.); 2 Clinic of Gynecology and Obstetrics, University Clinical Center Kragujevac, 34000 Kragujevac, Serbia; 3 Faculty of Medical Sciences, Department of Pharmacy, University of Kragujevac, 34000 Kragujevac, Serbia; tnikolict@gmail.com (T.N.T.); katarina.mih17@gmail.com (K.M.); nbarudzic@hotmail.com (N.J.); pindovic.bozidar@gmail.com (B.P.); 4 N.A. Semashko Public Health and Healthcare Department, F.F. Erismann Institute of Public Health, I.M. Sechenov First Moscow State Medical University (Sechenov University), 119435 Moscow, Russia; 5 Center of Excellence for Redox Balance Research in Cardiovascular and Metabolic Disorders, 34000 Kragujevac, Serbia; jovana_joksimovic@yahoo.com; 6 Department of Radiology, University Clinical Center Kragujevac, 34000 Kragujevac, Serbia; 7 Faculty of Medical Sciences, Department of Pathology, University of Kragujevac, 34000 Kragujevac, Serbia; smitrovic@fmn.kg.ac.rs (S.M.); spasojevicmarija89@gmail.com (M.S.); jecasavic998@gmail.com (J.S.); 8 Department of Pathology, University Clinical Center Kragujevac, 34000 Kragujevac, Serbia; 9 Federal State Autonomous Educational Institution of Higher Education I.M. Sechenov First, Moscow State Medical University of the Ministry of Health of the Russian Federation (Sechenov University), 119991 Moscow, Russia; 10 Faculty of Medical Sciences, Department of Physiology, University of Kragujevac, 34000 Kragujevac, Serbia; 11 Faculty of Medical Sciences, Department of Surgery, University of Kragujevac, 34000 Kragujevac, Serbia; gbalovic@gmail.com; 12 Center of Pediatric Surgery, University Clinical Center Kragujevac, 34000 Kragujevac, Serbia

**Keywords:** COVID 19, pregnancy, placental pathological lesions, neonatal oxidative stress, SARS-CoV-2 fetal transmission

## Abstract

**Background:** The impact of the SARS-CoV-2 viral infection during pregnancy on the fetus can be direct—transmitted through the placenta—and indirect—creating unfavorable conditions for the development of the fetus because of inflammation, micro-thrombosis, and hypercoagulation. Our study aimed to determine the types and frequency of pathohistological changes in placental tissue in SARS-CoV-2-positive pregnant women and to examine the possible role of oxidative stress in the prognosis of the delivery and its maternal and fetal complications. **Methods:** This prospective clinical study included 50 pregnant women divided into two groups, SARS-CoV-2 positive (COVID-19 group) and SARS-CoV-2 negative (control group), from who we collected demographic, clinical, obstetric, biochemical and pathologic data. Data about the newborn characteristics were also collected, which included anamnestic, clinical, and biochemical data. **Results:** The values of the superoxide anion radical and index of lipid peroxidation were significantly different in mothers concerning the presence of the SARS-CoV-2 infection, while the levels of the nitric oxide, index of lipid peroxidation, reduced glutathione, and superoxide dismutase were significantly different in the newborns depending on the SARS-CoV-2 infection. Newborn characteristics were similar between groups except for concentrations of IgM antibody. The incidence of pathohistological changes of the FVM type in the COVID-19 group of pregnant women was 46%, while in the control group, the incidence was 18%. **Conclusions**: This study confirmed the significant impact of the SARS-CoV-2 viral infection on maternal and fetal biochemical parameters and oxidative stress-mediated placental dysfunction. Future studies should be performed with more participants and follow-up neonatal development.

## 1. Introduction

WHO data show that the number of patients with COVID-19 from the beginning of the pandemic to August 2024 exceeds 776 million, with more than 6.95 million deaths [1,2].

Even after the WHO declared the end of the pandemic on 11 May 2023, COVID-19 remains one of the biggest challenges facing the health system and world economy because of its rapid spreading and unpredictable course.

SARS-CoV-2 is the pathogen responsible for the onset of COVID-19, belonging to the group of beta-type Coronaviridae [3,4]. Based on the structure, it is an enveloped RNA virus that shares a genetic sequence with some SARS viruses identified in bats and pangolins, indicating a zoogenic origin [5]. SARS-CoV-2 components are RNA and four structural proteins: spike (S) protein, nucleocapsid (N) protein, envelope (E) protein, and membrane (M) protein with a specific role in the pathogenesis of the disease [6].

The RNA of the virus is a template for the multiplication of viral RNA and proteins that are further released infecting new cells. The release of the virus into the bloodstream leads to an inflammatory reaction mediated by the release of cytokines and the consequent activation of the coagulation system [7,8]. The activation of monocytes as part of the acute phase response leads to damage to the endothelium, which is crucial for activating the procoagulant system within the body and forming immune-thrombosis.

Based on the severity of symptoms, COVID-19 can be asymptomatic, mild, moderate, severe, and critical [9]. The viral load, inflammation, and the state of the immune system also influence the severity of the clinical course [1]. Factors such as age, gender, associated diseases, and obesity are related to the severity of the disease and increased mortality [10]. SARS-CoV-2 infection also interferes with a woman’s reproductive health, negatively affecting ovarian function [11]. The physiological changes that accompany pregnancy could theoretically contribute to the development of more severe forms of COVID-19 in this group of patients [12]. Based on clinical experience and the conclusions of studies conducted during previous epidemics caused by coronaviruses such as Severe Acute Respiratory Syndrome (SARS) and Middle Eastern Respiratory Syndrome (MERS), patients infected during pregnancy are classified as patients with an increased risk of developing severe forms of disease and mortality [13,14]. There is a growing concern about possible neonatal and perinatal complications based on experience with ZIKA infection that is capable of crossing the placenta and causing fetal development damage [15]. Data from the US Centers for Disease Control and Prevention show that pregnant women have the same risk of death, but an increased risk of developing complications that require hospitalization and admission to the ICU compared to patients who are not pregnant [7].

Direct cytotoxicity of the virus and endothelial injury led to inflammatory changes in the complex functioning of the pregnant woman’s immune system. This system constantly changes to satisfy the demands of the growing embryo and, thus, the time of infection can be of great importance for the prognosis of the course of the disease, maternal immune response, release from the virus, and perinatal outcome [16].

The impact of viral infection on the fetus can be direct—transmitted through the placenta—and indirect—creating unfavorable conditions for the development of the fetus because of inflammation, micro-thrombosis, and hypercoagulation.

There are three modes of transmission of the virus to the fetus/neonate: direct/intrauterine transmission, intrapartum, and postpartum transmission.

Oxidative stress is one of the key factors in the pathogenesis of COVID-19 infection [17,18,19]. Oxidative stress is an impaired homeostasis in the body between oxidants and antioxidants, which leads to further disruption of the redox system and damage to molecules. Free radicals are highly reactive molecules with an excess of one or more electrons that easily interact with other molecules and lead to disruptions in the physiologically tuned system of a healthy organism. Excessive production of free radicals: reactive oxygen species (ROS), reactive nitrogen species (RNS), and reactive chlorine species (CRS) lead to direct peroxidation of membranes, structural proteins, enzymes, and nucleic acids, and can be neutralized under physiological conditions via an interaction with enzymatic and non-enzymatic antioxidants. In addition to endogenous (enzymatic and non-enzymatic antioxidants), there is also a group of exogenous (natural or synthetic antioxidants) that also have a role in binding unpaired electrons of free radicals and stabilizing them, and their cumulative effect is defined as total antioxidant capacity (TAS) [20,21,22,23].

During scientific attempts to determine the level of oxidative stress in the body, a whole panel of biochemical markers of OS were created, which, based on their origin, were divided into:

Molecules that interact with free radicals, such as:

- Products of lipid peroxidation: malondialdehyde, acrolein, isoprostanes),
- Protein oxidation products: (carbonyl protein, tyrosine group),
- DNA oxidation products (8-hydroxy-2deoxyguanosine),
- Protein stress reactions, new biomarkers (microRNAs)
  Molecules that belong to the antioxidant protection system, such as
- Enzymes (myeloperoxidase),
- Glutathione level
- Circulating antioxidants (CAT, GPX, SOD) [22,24,25,26]

Pregnancy itself represents a state of increased oxidative stress due to the intense metabolic and immunological alterations necessary for the complex process of growth and development of conception products. The main source of ROS in pregnancy is the placenta, where these molecules are key factors in the complex system responsible for intercellular signaling, transcription, adaptive homeostasis, apoptosis, and host defense through phagocytosis [27,28,29,30]. Viral infections affect the excitation of oxidative stress of the host through numerous mechanisms such as excessive cytokine production, activation of the complement system, release of lipid mediators, and lipid peroxidation during the interaction of the body’s defense cells with viral pathogens [31,32].

Examining the changes in the placenta provides us with a unique opportunity to uncover the mechanisms by which the virus affects pregnancy and the fetus. In addition to its nutritional, excretory, oxygenation, and hormonal function, the placenta has an important protective role and is the main barrier to bacterial and viral infections in the fetus. This role predisposes the placenta to pathological changes under the direct influence of the SARS-CoV-2 virus and the consequences of systemic disease and coagulation changes. Based on the Amsterdam classification system, placental lesions could be defined as maternal vascular malperfusions, fetal vascular malperfusions, acute chorioamnionitis, and villitis of unknown etiology [33].

We hypothesized that:

1. There is a statistically significant difference in the frequency of pathohistological changes in placental tissue in patients with confirmed SARS-CoV-2 infection during pregnancy compared to the control group.
2. Vertical transmission of SARS CoV-2 from mother to fetus is possible.
3. Compared to the control group, there is a statistically significant increase in maternal and umbilical cord blood biomarkers of oxidative stress in patients infected with SARS-CoV-2 during pregnancy.
4. Patients with more pronounced pathohistological changes in placental tissue have significantly elevated levels of oxidative stress markers.
5. There is a statistically significant difference in the neonatal outcome of newborns of mothers with COVID-19 compared to the control group.
6. Certain clinical parameters such as gestational age, sex of the newborn, and maternal age significantly affect the level of oxidative stress markers in the newborn.

This study aims to:

1. To determine the types and frequency of pathohistological changes in placental tissue in patients in whom SARS-CoV-2 infection was confirmed during pregnancy and compare them with the control group
2. Determine from the plasma and lysate of erythrocytes of the mother’s blood immediately before delivery and the blood of the newborn from the umbilical cord the values of the parameters of the antioxidant protection system, as well as pro-oxidants: lipid peroxide index measured as TBARS, nitric oxide in the form of nitrite (NO_2_^−^), superoxide anion radical (O_2_^−^) and hydrogen peroxide (H_2_O_2_), catalase (CAT), superoxide dismutase (SOD), reduced glutathione (GSH).
3. Determine the neonatal outcome: Apgar score, body weight of the newborn, admission to the intensive care unit, and length of stay in the neonatology department and ICU

## 2. Materials and Methods

### 2.1. Ethical Concerns

Following the Good Clinical Practice and revised Helsinki Declaration, this study was designed as a case-series clinical study. The Ethical Committee of Clinical Center Kragujevac approved the study number: 01/21-18 from 28 January 2021. Written informed consent was obtained from all participants for research and publication before inclusion in the study.

### 2.2. Participants

This prospective clinical study included pregnant women who gave birth from February 2021 to March 2022 at the at the Gynecology Clinic at University Clinical Center Kragujevac, aged between 18 and 43 years old, from Central West Serbia, followed during the period from the diagnosis of a positive SARS-CoV-2 test to discharge from the obstetrics department after delivery and discharge of the newborn from the Department of Neonatology at University Clinical Center Kragujevac, Serbia.

### 2.3. Protocol of Study

Two cohorts were derived from the population of pregnant women who were delivered at the OB/GYN Clinic of the University Clinical Center in Kragujevac: women who had a positive result for SARS-CoV-2 (by RT-PCR method and/or rapid antigen test) during the pregnancy. The control cohort were woman who had a negative result on a rapid antigen or RT-PCR SARS-CoV-2 test on their admission day to the Obstetric Clinic for delivery, who were asymptomatic during hole pregnancy, and who had no IgG and IgM SARS-CoV-2 antibodies detected on serological blood test. The main inclusion criteria were positive RT-PCR and/or rapid antigen tests for SARS-CoV-2 during the pregnancy. Exclusion criteria were positive personal history of thromboembolic diseases, thrombophilia, antiphospholipid syndrome, diabetes mellitus, systemic lupus erythematosus, hypertensive disorders in pregnancy, Rhesus factor incompatibility, ongoing anticoagulant therapy, tobacco smoking during pregnancy, and vaccination against COVID 19. Within the cohort of pregnant women who tested positive for the presence of SARS-CoV-2 during pregnancy concerning the time of gestation in which the infection occurred, three subgroups of patients were distinguished, within whom the impact of COVID-19 on placental tissue was monitored in parallel:

1. Patients who were diagnosed with SARS-CoV-2 in the first trimester of pregnancy (from pregnancy to the end of week 13 of gestation)
2. Patients who were diagnosed with SARS-CoV-2 in the second trimester of pregnancy (from 14 to the end of week 27 of gestation)
3. Patients diagnosed with SARS-CoV-2 in the third trimester of pregnancy (28 to 42 weeks of gestation)

### 2.4. Sampling and Collecting Data

We reviewed the electronic medical record for each subject and recorded demographic, clinical, obstetric, laboratory, and pathological data. At that time, none of the patients in either group were vaccinated against COVID-19. Blood samples were collected from those two groups following delivery, namely: maternal peripheric blood and the umbilical cord blood for oxidative stress biomarkers testing, and placental tissue (a section of the entire thickness of the placenta with the membranes and one section of the umbilical cord for pathohistological analysis, fixed for 48 h in a formalin solution to eliminate the infectivity).

### 2.5. Determination of Oxidative Stress Markers

We collected venous blood from each participant, maternal peripheric blood, and umbilical cord blood for oxidative stress biomarkers testing within 3 min following delivery. Oxidative stress biomarkers were assessed spectrophotometrically from plasma samples (Shimadzu UV 1800, Tokyo, Japan), the index of lipid peroxidation, determined as thiobarbituric acid-reactive substances (TBARS), nitrites (NO_2_^−^), levels of superoxide anion radical (O_2_^−^), and hydrogen peroxide (H_2_O_2_).

#### 2.5.1. Determination of TBARS

The plasma sample was incubated with 1% thiobarbituric acid in 0.05 NaOH for 15 min at 100 °C and measured as TBARS at 530 nm. As a control, distilled water was used as a blind probe [34].

#### 2.5.2. Nitrite Determination (NO_2_^−^)

Rapidly breaking down nitric oxide (NO) produces stable nitrite/nitrate compounds determined spectrophotometrically at 543 nm using Griess’s reagent according to Green’s method. Sodium nitrite was used as the reference standard to calculate the nitrite levels [35].

#### 2.5.3. Superoxide Anion Radical Determination (O_2_^−^)

Superoxide anion radical quantities were determined using an assay mixture containing nitroblue tetrazolium. The measurement was carried out at 530 nm on the wavelength. The blank control was performed using distilled water [36].

#### 2.5.4. Hydrogen Peroxide Determination (H_2_O_2_)

Horseradish peroxidase was utilized to accelerate the oxidation of phenol red by H_2_O_2_, which was used as a basis for the H_2_O_2_ measurement. 800 µL of freshly created phenol red solution was used to precipitate 200 µL of perfusate, and 10 µL of freshly made (1:20) horseradish peroxidase was added next. Distilled water was utilized as the blank probe. At 610 nm, the level of H_2_O_2_ was detected [37].

#### 2.5.5. Catalase Activity Determination (CAT)

The analysis of catalase activity was carried out based on the Aebi method [38].

First, we diluted the hemolysate with distilled water in a ratio of 1:7. An amount of 100 μL of the hemolysate sample was then mixed with an equal volume of ethanol along with 50 μL of CAT buffer and 1000 μL of 10 mM H_2_O_2_. The catalase activity was then determined spectrophotometrically at a wavelength of 230 nm [38].

#### 2.5.6. Superoxide Dismutase Activity Determination (SOD)

The activity of superoxide dismutase was carried out using the Beutler method. In Eppendorf, we first mixed [39] 100 μL of hemolysate with 1 mL of carbonate buffer, then processed the sample into a vortex and finally added 100 μL of adrenaline. Spectrophotometry was performed at a wavelength of 470 nm [39,40].

#### 2.5.7. Reduced Glutathione Concentration Determination (GSH)

The concentration of reduced glutathione (GSH) was determined using the Beutler method. The method principle is based on the oxidation reaction of GSH with 5,5-dithio-bis-6,2-nitrobenzoic acid. Spectrophotometric measurements were made at a wavelength of 412 nm [39].

### 2.6. Pathohistological Analysis of Placental Tissue

Samples were also collected according to the recommendations of the Amsterdam Consensus [41]. Six samples were taken: first a sample of the amniotic sheath from the rupture site to the edge of the placenta; a sample of the cross-section of the umbilical cord, one from the fetal and the other about 5 cm from the uterine insertion; and three more sections of the full-thickness placental disc cut with a scalpel, covering the uterine and fetal sides, taken from the central two-thirds of the surface of the placenta and one from the insertion site.

Tissue processing: dehydration, rinsing, and impregnation were carried out using a tissue processor and then molded into paraffin blocks. Then, using a rotating microtome (Leica RM2135, Tokyo, Japan), we cut the tissue into samples 5 μm thick, at room temperature, which were then immersed in a water bath at a temperature of 400 °C. The cross-sections processed in this way were applied to the subject glasses (Superfrost-OT Plus microscope slides) for microscopy.

#### Staining Hematoxylin–Eosin Technique

To de-paraffin the tissues, it was necessary to first subject the glasses to heating to +56 °C and then to spontaneous cooling. After that, we immersed them in xylol consecutively twice for 5 min each. Once we deparaffinated the tissues, we proceeded to rehydrate the tissues by immersing the glasses in solutions with decreasing concentrations of ethyl alcohol and then evaporating them with distilled water. The next step was staining with Mayer solution (Sigma Aldrich, St. Louis, MO, USA) for 10 min, after rinsing with distilled water and staining with an alcohol solution of eosin (Sigma Aldrich, USA) for the next 2 min. A dehydration process was then carried out, which involved submerging the glass by increasing concentrations of ethyl alcohol and then illuminating it with two xylol solutions, each for 5 min [42]. The tiles prepared in this way were then protected by cover glass after treatment with a medium covering and dried at room temperature. The analysis was carried out independently by two experienced pathologists on an Olympus BX51 light microscope, Tokyo, Japan. In the case of inconsistent results, the attitude of the third pathologist was decisive.

### 2.7. Statistical Analysis

All data are presented in the form of tables and graphs. Statistical analysis was done using the descriptive (mean, standard deviations and errors, and analytical tests, frequency, range) and analytical tests (Student *t*-test, Chi-square test). The statistical threshold was set at 0.05. All analysis was done in SPSS version 26 for Apple. Inc. (Cupertino, CA, USA).

## 3. Results

### 3.1. Basic Demographic and Anamnestic Data of Study Group

This study included 50 pregnant women divided into two groups, SARS-CoV-2 positive (COVID-19 group) and SARS-CoV-2 negative (control group). In the first group, the mean age was 30.61 ± 4.72, and in the negative groups, the mean age was 31.41 ± 4.65. The mean gestational week at delivery was 39.19 ± 0.98 in SARS-CoV-2 positive women, and 39.42 ± 1.26 in the group SARS-CoV-2 negative. Based on the severity of symptoms 41.38% of patients had mild symptoms, and 58.62% had moderate symptoms.

### 3.2. Characteristics of the Newborns at Delivery According to the Presence of SARS-CoV-2 Infection in Mothers

Table 1 presents the mean values of the newborn characteristics at delivery. Most of them were similar in both groups, except for the concentrations of IgM antibody, where we found that newborns from the COVID group of mothers had significantly higher levels of these inflammatory markers in comparison with the SARS-CoV-2 negative group (as shown in Table 1).

### 3.3. Analysis of the Oxidative Stress Levels According to the Presence of SARS-CoV-2 Infection

**Figure 1:**
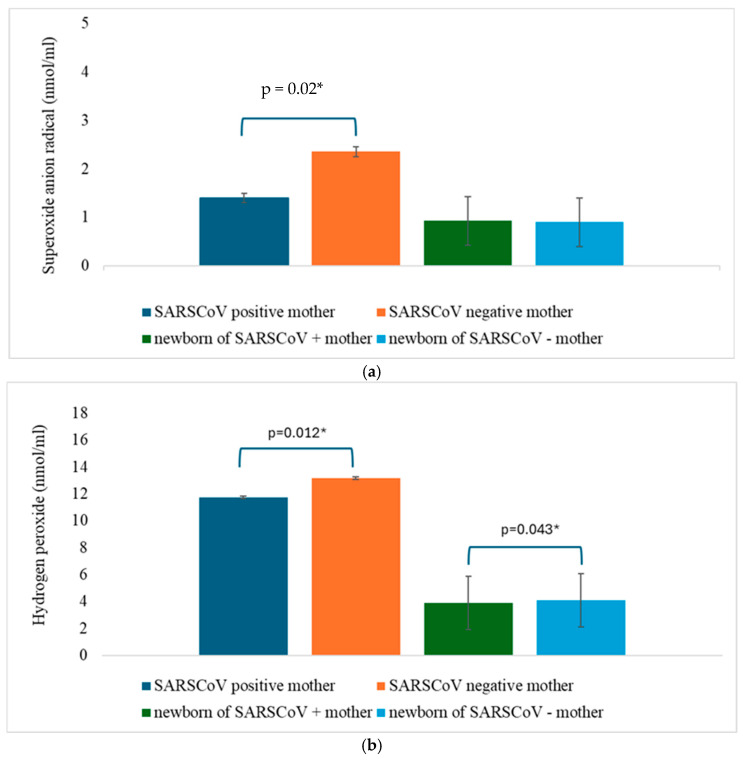

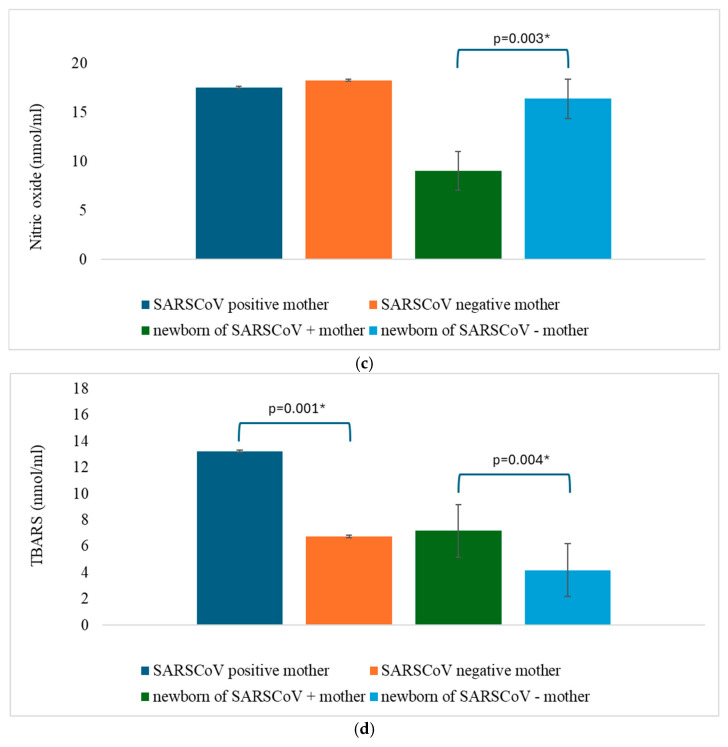
(**a**) The mean concentration of the plasma superoxide anion radical (O_2_^−^). Results are presented as the mean plus standard deviations. Statistical analysis was done using the Student *t* test. The asterisk (*) represents the statistically significant value (*p* < 0.05). (**b**) The mean concentration of the plasma hydrogen peroxide (H_2_O_2_^−^). Results are presented as mean plus standard deviations. Statistical analysis was done using the student *t* test. The asterisk (*) represents the statistically significant value (*p* < 0.05). (**c**) The mean concentration of the plasma nitric oxide (NO^−^). Results are presented as mean plus standard deviations. Statistical analysis was done using the Student *t* test. The asterisk (*) represents the statistically significant value (*p* < 0.05). (**d**) The mean concentration of the plasma index of lipid peroxidation (TBARS). Results are presented as mean plus standard deviations. Statistical analysis was done using the Student *t* test. The asterisk (*) represents the statistically significant value (*p* < 0.05).

**Figure 2:**
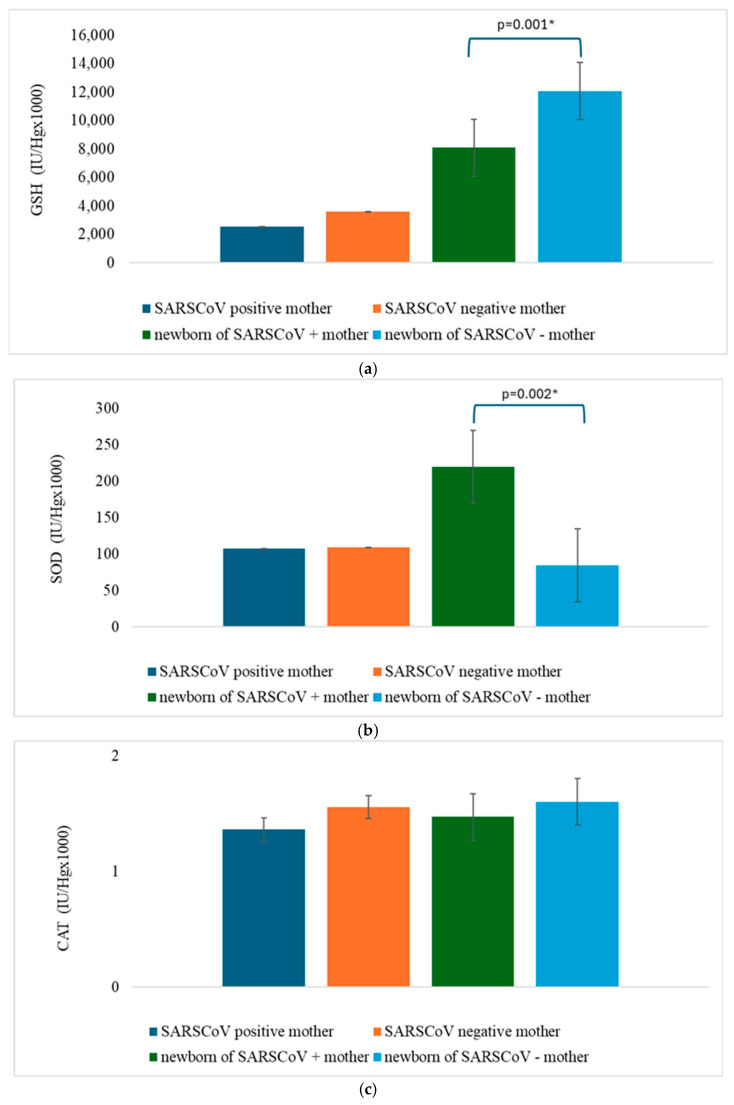
(**a**) The mean activity of the hemolysate reduced glutathione (GSH). Results are presented as the mean plus standard deviations. Statistical analysis was done using the Student *t* test. Asterisk (*) represents the statistically significant value (*p* < 0.05). (**b**) The mean activity of the hemolysate superoxide dismutase (SOD). Results are presented as mean plus standard deviations. Statistical analysis was done using the Student *t* test. Asterisk (*) represents the statistically significant value (*p* < 0.05). (**c**) The mean activity of the hemolysate catalase (CAT). Results are presented as mean plus standard deviations. Statistical analysis was done using the Student *t* test.

According to the presence of the SARS-CoV-2 infection, the mean values of redox status parameters are presented in the form of Figure 1a–d and Figure 2a–c. The values of the superoxide anion radical and index of lipid peroxidation were significantly different in mothers concerning the presence of the SARS-CoV-2 infection, while the levels of the nitric oxide, index of lipid peroxidation, reduced glutathione, and superoxide dismutase were significantly different in the newborns depending on the SARS-CoV-2 infection (Figure 1 and Figure 2, Table 2). Other parameters were not significantly changed (Figure 1 and Figure 2, Table 2).

### 3.4. The Histopathologic Lesions in the Placenta Concerning the Presence of SARS-CoV-2 Infection and the Timing of Infection

The incidence of pathohistological changes of the FVM type in the COVID-19 group of pregnant women was 46%. In contrast, in the control group, they were represented in 18% of placentas, and the incidence of MVM in the COVID group was 32%, and in the experimental group, this was 18%. According to the moment of infection in the first trimester, pathohistological changes were registered in only 3% of patients infected in the first trimester. In contrast, they were more prevalent when the infection occurred during the second and third trimesters in 44.4% and 47% of patients in the COVID group. Inflammatory lesions in placental tissue were detected in 14.81% of placentas in the COVID group and only 4.5% of subjects in the control group. A total of 70% of all placental lesions were detected in patients with a moderate/severe presentation of COVID-19. Table 3 presents mothers’ main morphometrical placental characteristics regarding the presence of COVID-19. The results are presented as the frequency of the observed entity as a percentage (%) (Table 3).

### 3.5. Neonatal Outcome

In the COVID-19 group, 62% of newborns were girls, 38% were boys, and in the control group, 41% were girls and 59% were boys.

The mode of delivery was vaginal birth in 68%, and C-section in 32% in the COVID-19 group, and vaginal birth in 45%, and C-section in 55% in the control group. Newborns of mothers from the COVID group had a similar mid-length of hospitalization—4.37 ± 1.1 days—as newborns from the control group, who had a mid-length of hospitalization 4.22 ± 2.72 days.

The length of newborn stay in the neonatal ICU was 3.51 ± 1.08 days in the COVID group and 2.22 ± 1.39 days in the control group. Using a t-test for two independent samples we noticed a significant difference in the length of hospitalization in the NICU between the two groups (*p* ≤ 0.0006).

Newborns from COVID-positive mothers were tested with nasopharyngeal swabs for RT-PCR SARS-CoV-2 test in the first 24 h post-delivery. Only one positive result was registered in newborns.). A total of 42.8% of newborns from the COVID group mothers had positive SARS-CoV-2 IgM antibody results, and in the control group there were no positive SARS-CoV-2 IgM antibody results.

## 4. Discussion

This prospective clinical study aimed to analyze the differences between basic, clinical, anamnestic, and biochemical data among pregnant women who were affected by COVID-19 or not affected. The special purpose of this research lies in the evaluation of the effects of the SARS-CoV-2 infection on fetal and newborn characteristics. Based on previous findings and literature data, for the first time, this study evaluated the pathohistological changes in placental tissue among COVID-19 women who gave birth.

In the COVID group predominant mode of delivery was vaginal birth, except in a group of pregnant women with active COVID infection during delivery. Although the C-section birth rate was high (32%) it was significantly lower than previous studies showed [43,44,45]. The leading indications for C-section in the COVID group were previous delivery completed by C-section, fetal distress during labor, and malpresentation, similar to the control group. Our study showed that there was no statistically significant increase in the number of cesarean sections in patients with COVID-19 during pregnancy. Studies dealing with this issue had contradictory results, and some investigators have shown an increased risk of operative termination of labor in COVID-19 patients [46,47,48], but others have shown that the mode of delivery does not depend on COVID-19 infection in pregnancy [49].

No significant differences in neonatal outcomes were found between COVID-19 and the control group regarding birth weight, length, and head circumference. Some similar studies showed associations between COVID-19 symptoms and birth weight, the risk of prematurity, and ventilation in the early neonatal period [50]. The hospitalization length for newborns was similar in both groups. No significant decrease in hospitalizations was noted in our study, despite expectations because of the suspension of elective admissions, similarly as Gaetano’s study has shown [51].

Neonatal ICU hospitalization length was 3.51 ± 1.08 days in the COVID group and 2.22 ± 1.39 days in the control group. Our findings support expert recommendations about the necessity of multidisciplinary teams available in hospitals that take care of COVID-19-positive and suspected mothers and their infants [52]. Additionally, a neonatal intensive care unit is mandatory for hospitals that take care of the delivery of those patients, and there is no clear evidence about possible direct transmission of SARS-CoV-2 from mother to infant.

Pregnancy and placental growth and development are accompanied by numerous changes due to the balance of the ROS and the antioxidant protection system, which is also involved in the complex mechanisms of cell signaling. The development of the placenta begins with the insertion of a fertilized blastocyst and the primary mode of transport through the placenta is diffusion conditions of low partial pressure of O_2_, which is necessary for the normal growth and development of the embryo and the prevention of the formation of ROS [53]. As pregnancy progresses from the periphery of the placenta to the center, there is a gradual opening of the occluding blood vessels and a gradual increase in the partial pressure of oxygen within the intervillous space due to the continuous flow of oxygenated blood. This process takes place at the end of the first trimester and is responsible for the shift in the partial pressure of oxygen in the intervillous space [54]. When there is a disturbance at any level of placentation, the result is a sudden increase in oxygen pressure within the intervillous space and the consequent formation of ROS [55]. A gradual increase in O_2_ pressure allows the placenta to adapt to the new conditions of ROS formation by developing an antioxidant protection system (primarily by producing GPx and CAT) [56]. To counteract the effect of ROS (O_2_^−^, NO, H_2_O_2_, ONOO^−^) in the cells of the cytotrophoblast and stroma, a protection system is developed that includes the enzymes MnSOD, CuSOD, ZnSOD. However, when the level of ROS production exceeds the antioxidative capacity of the placenta, protein, lipids, and DNA damage occurs with consecutive cell damage and death [53,56].

The determination of individual biomarkers of oxidative stress in placental pathology has no scientific or clinical significance, but a comparative examination at birth showed a positive correlation between elevated values of OS biomarkers in the mother’s blood and blood from the umbilical cord of the newborn [57]. Placental ischemia, resulting from SARS-CoV-2 infection, or systemic hypoxia and inflammation, leads to a disturbance in the balance of ROS and antioxidant protection and the onset of oxidative stress. OS is responsible for damage to proteins, lipids, and DNA. The placenta has mechanisms to counteract oxidative stress to reduce tissue damage, such as the production of NO, a potent vasodilator and antioxidant [58].

Our study showed that levels of TBARS were significantly increased in mothers of the COVID-19 group (*p* = 0.002) compared with mothers of the control group, possibly because of lipid membrane damage caused by the SARS-CoV-2 virus. O_2_^−^ levels were significantly higher in mothers from a control group and H_2_O_2_ levels were significantly higher in mothers and neonates from a control group, which corresponds with the mode of delivery. SC was more frequent in the control group 55% and 32% in the COVID-19 group. The method of delivery could significantly affect the elevated level of biomarkers OS in the newborn, where these values are significantly increased in prolonged vaginal delivery and emergency cesarean section, as well as in stimulated oxytocin delivery. [20,59,60,61]. Additionally, the leading indication for C-section in the control group was non-reassuring fetal status and previous C-section.

Levels of NO were significantly lower in newborns of the COVID-19 group in our study, indicating that the placentas of COVID mothers could not sufficiently counterbalance the production of ROS after and during SARS-CoV-2 infection. Increased levels of SOD in COVID-19 group newborns suggested that the placenta developed an enzymatic oxidative damage defense system.

The effect of the oxidative stress of the mother on the oxidative stress of the fetus depends on the antioxidant capacity of the newborn, which is determined genetically, depending on the sex of the fetus, the maturity of the fetus, and the age of the mother [20].

Studies focused on the effect of oxidative stress on placental tissue depending on the timing of infection in pregnancy have shown that changes within the scinciotroblast dominate the first trimester compared to the cytotrophoblast. This is reflected in a decrease in the surface area of microvilli and a decrease in mitochondria, with a very low amount of antioxidants detected [62]. A factor responsible for the onset of oxidative stress during the second and third trimesters is the intermittent flow of maternal blood within the villus, which produces ischemic reperfusion damage [63]

Some studies have proposed that the determination of different types of OS biomarkers from the umbilical cord, placenta, and maternal blood could have implications in the prediction of placental pathology and clinical symptomatology [58,64]. Our study showed that TBARS could have clinical implications in identifying high-risk COVID-19 pregnancies requiring closer monitoring and neonatal care with pediatric follow-ups watching neonatal development. Other OS biomarkers in our study were more dependent on the mode of delivery and placental ability to counterbalance the oxidative stress.

Besides oxidative stress damage, a potential pregnancy hazard is the transmission of the virus to the fetus. There are a few possible maternal/fetal (neonatal) infection methods. Fetal infection can occur in utero by direct transplacental transmission. The other possible way is intrapartum infection (neonatal contact with cervical or vaginal secretions or blood), and the third way is after birth via breastfeeding or direct maternal-neonatal contact [65]. All patients with active COVID newborns were tested with a SARS-CoV-2 RT PCR test, only one was positive. Our study found that 42.8% of newborns from the COVID group mothers had positive IgM antibody findings. A similar study performed by Maranto showed IgM positive in 2.4% of newborns [50]. Initial studies focused on direct transmission showed that there was no convincing evidence of this mode of infection; however, at the end of the pandemic, worldwide pandemic meta-analyses showed that about 8.8% of newborns of COVID-19-positive mothers at birth had a positive SARS-CoV-2 PCR or serological test obtained after taking a nasopharyngeal swab [66,67]. The method of determining direct transmission to the fetus is also a subject of scientific doubt. In adults, the respiratory tract is the primary site of infection and the highest concentration of the virus, and the analysis of samples is performed through a nasopharyngeal or oropharyngeal swab. In a newborn, in addition to this method, it is proposed to do the serological determination of the concentration of specific IgM antibodies to SARS-CoV-2, concerning the unknown site of primary infection [68,69]. IgM antibodies as part of the immune response to a specific viral infection, unlike IgG antibodies, cannot cross the placental barrier and can be useful in determining potential direct transmission to the fetus, but the weakness of this method is the existence of a cross-reaction and a false positive result, and the PCR test of the nasopharyngeal/oropharyngeal mucosa is the gold standard for determining the presence of SARS-CoV-2 infection in the newborns [70]. Studies focused on direct transmission have also used RNA Scope and in-situ hybridization in scinciciotrophoblast cells, but the main problem with interpreting the results is the inconsistency of the results of the available tests. The sensitivity of the RT-PCR test is highest from the bronchial lavage sample (93%), and significantly lower from the nasopharyngeal swab and feces (63% and 29%, respectively), but as it is invasive, this method is not suitable for testing pregnant women and newborns [71].

Investigating routes of possible fetal or neonatal transmission should provide sufficient information for future decisions considering the obstetric way of delivery, neonatal care, isolation of neonates, or safety of breastfeeding [72].

During this interaction of viruses and the host, if the overproduction of ROS exceeds the antioxidant capacity of the placenta, tissue damage may occur [31,73]. Maternal vascular malperfusions (MVM) were the most frequent finding of some studies, while others have reported fetal vascular malperfusions (FVM) as a predominant finding, diagnosed according to the Amsterdam criteria based, on the side of the placenta on which they occurred [45,74,75,76,77]. (FVM) Fetal vascular malperfusions are specific lesions of the placenta that arise from the obstruction of fetal blood flow. They can originate from the umbilical cord (hyperkoyling of the umbilical cord), from hypercoagulability, hypoxia, and inflammation-mediated endothelial damage (acute chorioamnionitis with chorionic vasculitis or funisitis and chronic villitis) [78,79,80,81,82].

According to the Amsterdam criterion, FVM is represented by the following findings: thrombosis, segmental avascular villi, villous stromal-vascular karyorexa, vascular intramural fibrin deposition, blood vessel obliteration/fibromuscular sclerosis and vascular ectasia [79]. MVM encompasses a spectrum of macroscopic and microscopic changes in the placenta. These changes are the result of ischemic (hypoxic) damage and oxidative stress [83]. Macroscopic changes are placental hypoplasia (weight < 10th percentile), narrowed umbilical cord (diameter less than 0.8 cm at term), infarction, and retroplacental hemorrhage [41,79,84]. Microscopic lesions of the placenta include distal villi hypoplasia and accelerated villi maturation [41,79,84].

In addition to vascular changes, some studies have also proven the presence of inflammatory histopathological changes within the placentas of patients infected with COVID. Chronic villitis can be a consequence of viral infections, but in most cases, the etiology has not been identified [85]. Several studies have examined macroscopic and microscopic histopathological changes within the placental tissue in patients infected during pregnancy, but the results are incoherent [86,87,88,89,90,91,92]. Some studies even demonstrated no specific placental pathological findings in COVID patients regardless of the timing or severity of the disease [93,94]. The weaknesses of these studies are represented by the small number of samples, the inconsistency in the classification of histopathological changes, confounding factors such as chronic diseases in pregnancy and lifestyle habits that can have a significant impact on the result, as well as the choice of the control group.

**Figure 3:**
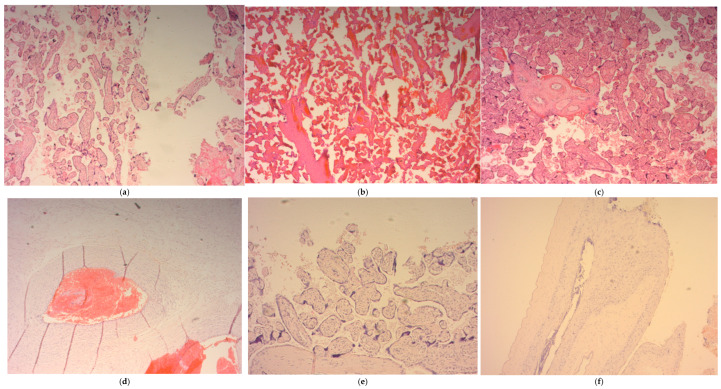
The representative pathohistological findings in placental tissue samples among the study population; (**a**,**b**) distal villous hypoplasia-long villi with a wide intervillous space; (**c**) the vascular ectasia-enlarged luminal diameter of the chorionic vessel; (**d**) villus vessel thrombosis-organized intraluminal thrombus; (**e**) avascular villus-lose of vessels with preservation of trophoblast; (**f**) chorioamnionitis-numerous polymorphonuclear leukocytes infiltrate edematous fetal membranes; magnification 100×.

Our study showed that placental pathology findings differ significantly between COVID-19 and the control group. We have found MVM lesions in 32% of the placentas of the COVID group and 18% of patients in the control group (Figure 3a,b). FVM lesions were present in 46% of the placentas of patients in the COVID group and 18% of patients in the control group (Figure 3c–e). Previous studies showed unequivocal results of placental findings, some demonstrated FVM in 30% and the other only 8% of placentas examined [76,95]. The most frequent findings in placental pathology of COVID patients in our study were: the obliteration of blood vessels, avascular villi, retroplacental hemorrhage, and accelerated villus maturation. Other studies’ most frequent pathophysiological findings were fibrin deposition, microcalcification, thrombus (Figure 3d), avascular villi (Figure 3e), infraction, and villous edema (Figure 3f) [96].

Previous observational studies dealing with the problem of MVM have shown that these changes were asymptomatic, but the cumulative effect was reflected in placental hypoperfusion leading to adverse perinatal outcome and fetal complications and is associated with preeclampsia and intrauterine growth retardation (IUGR) [97,98,99,100]. IUGR is a fetus that does not reach its genetic potential during intrauterine growth and development and is present in as many as 15% of pregnancies [101].

FVM lesions are associated with abnormalities of the neonatal central nervous system, IUGR, fetal cardiac abnormalities, intrauterine fetal death, and stillbirth [65,74,82,102].

Impaired placental function is responsible for short and long-term adverse outcomes for the child and mother. Short-term consequences require urgent intervention in the antepartum or peripartum period. Placental pathologic lesions could be a valuable clue in discovering long-term consequences that require special attention [103]. However, pathological reports of placental tissue should be available not only to obstetricians but more importantly to pediatricians who should be aware of potential risks for a child’s long-term developmental consequences [104].

Besides the evident difference in the frequency of placental lesions between SARS-CoV-2 and the control group in our study, there was no evidence of a significant difference in fetal and neonatal outcomes except in the newborns’ length of stay in the NICU. There is evidence that fetuses exposed to SARS-CoV-2 influence intrauterine have a tenfold increased risk for neurodevelopmental delay [105], so further follow-up should be warranted for those neonates. Neurodevelopmental disorders are not easily recognizable so an adequate timeframe should be set to follow development through early childhood, preschool, and school-age [103]. Given that the growth and development of the placenta is a dynamic process controlled by genetics and immunology, the moment when COVID-19 infection occurs during pregnancy can be an important factor in histopathological changes and maternal and fetal outcomes. Some studies showed that the timing of infection is crucial for developing pathophysiological placental lesions [74].

Genetic abnormalities are associated with placental lesions and no favorable neonatal outcome, so future studies should take into consideration the performance of non-invasive prenatal diagnosis tests as a reliable alternative to invasive methods in reviling genetic abnormalities in participants’ offspring [106].

At the beginning of our study, COVID-19 vaccines were not available in our county, and during the study when vaccination started there were no clear recommendations for vaccination during pregnancy. Serbian Government Health Department recommended vaccination during pregnancy in March 2022 when the collection of material for our study was completed. They recommended the vaccination of all women of fertility age without the need to postpone pregnancy and vaccination during the whole period of pregnancy with the Pfizer-BioNTech vaccine [107] Even then, there was a low vaccine acceptance in the population of pregnant women in Serbia. Vaccination acceptance is dependable on the patient’s education level, perception of barriers to vaccines, but also gynecologist advice [108], so future studies should provide more adequate information for doctors and patients.

Future studies should consider the vaccinal status against SARS-CoV-2, and the possible influence of vaccines on placental changes and neonatal and maternal outcomes. Future studies should also determine the variant of the SARS-CoV-2 virus because some studies showed differences in fetal and maternal outcomes depending on the presence of variant Alpha, Delta, or Omicron [109].

Besides potential pregnancy hazards originating from placental pathological lesions and increased oxidative stress, there is also increased psychological stress influencing the mental health of pregnant women during the COVID-19 pandemic. The increased number of suicides and self-harming was noticed during the pandemic [13] Some studies showed a relationship between neuroticism and a fear of COVID-19, and neuroticism is also a predictor of depression [110]. Patients from this group of high-risk pregnant require continuous psychological support during this delicate lifetime moment that should even extend beyond childbirth [111].

Our study’s limitations include the small number of participants and the fact that it was unblinded for pathologists, so there is potential bias.

## 5. Conclusions

Based on the previous observation, in the summary we can conclude the following issues:

1. The types and frequency of pathohistological changes in placental tissue in patients with SARS-CoV-2 confirmed infection during pregnancy differs from the control group. FVM is found to be the most frequent placental lesion in the COVID-19 group. Additionally, there were no significant differences noticed in neonatal outcomes, and delayed consequences are possible for those neonates, so more frequent follow-ups should be warranted for children whose mothers were SARS-CoV positive during pregnancy.
2. Neonates of SARS-CoV-2 mothers had a longer NICU hospitalization length, so the approach to those high-risk pregnancies should be multidisciplinary with mandatory NICU in hospitals that provide medical care and delivery for SARS-CoV-2 positive patients.
3. Values of the parameters of the antioxidant protection system, as well as pro-oxidants: (lipid peroxide index measured as TBARS, nitric oxide in the form of nitrite (NO_2_^−^), superoxide anion radical (O_2_^−^), hydrogen peroxide H_2_O_2_, superoxide dismutase (SOD), reduced glutathione (GSH)) significantly differed between the COVID-19 and control group samples. T-BARS is a valuable biomarker of possible SARS-CoV-2 placental damage. It could help to identify high-risk COVID-19 pregnancies that require special attention regarding delivery and neonatal care.
4. Other OS biomarkers in our study were more dependent on the mode of delivery and placental ability to counterbalance oxidative stress.
5. Neonatal outcome: Apgar score and body weight of the newborn did not differ significantly between newborns from mothers of COVID-19 and the control group. COVID-19 patients do not have higher obstetrics potential for a C-section or instrumental delivery.
6. Placental pathology reports should be available to pediatricians because they could be valuable in identifying neonates with a risk for long-term developmental consequences.

COVID-19 pregnancies exhibited an increase in histopathological abnormalities of the placenta, namely vascular and inflammatory changes of unknown etiology, as well as disturbing the redox status of mothers and newborns. Future studies investigating the specific influence of SARS-CoV-2 on placental tissue and newborn outcomes are needed, with more participants considering the vaccinal status of the patient, variant of SARS-CoV-2, with longer follow-ups for newborns, following neonatal and child development through early childhood, preschool, and school age.

## Figures and Tables

**Table 1:** The mean values of the parameters related to the newborn at delivery according to the presence of SARS-CoV-2 infection. Results are presented as mean plus standard deviations. Statistical analysis was done using the Student *t* test.

Parameters		Gestational Age in Weeks at Delivery	APGAR Score 1′	APGAR Score 5′	Baby Body Weight (g)	Baby Body Length (cm)	Head Circumference (cm)	IgM Antibody (g/L)	Placenta Weight (g)
SARS-CoV-2+	Mean	39.19	8.96	9.10	3359.17	48.75	34.38	3.42	608.26
	SD	0.98	0.62	0.62	491.69	2.25	1.58	3.25	129.81
SARS-CoV-2-	Mean	39.42	9.23	9.19	3534.09	50.27	35.23	0.30	577.37
	SD	1.26	0.61	0.60	404.35	2.12	1.38	0.27	143.37
*p* value		*p* = 0.676	*p* = 0.556	*p* = 0.780	*p* = 0.465	*p* = 0.681	*p* = 0.899	*p* = 0.000 *	*p* = 0.322

A symbol asterisk (*) represents the *p* values less than 0.05.

**Table 2:** Data from statistical analysis using the Student T test in comparison with two groups of concentrations of pro-oxidative markers antioxidative enzymes. Asterisk (*) represents the statistically significant value (*p* < 0.05).

Comparison	Mothers	Newborns
	SARS-CoV-2+ vs. SARS-CoV-2−	SARS-CoV-2+ vs. SARS-CoV-2−
O_2_^−^	*p* = 0.002 *	*p* = 0.113
H_2_O_2_	*p* = 0.012 *	*p* = 0.043 *
NO^−^	*p* = 0.509	*p* = 0.003 *
TBARS	*p* = 0.001 *	*p* = 0.004 *
GSH	*p* = 0.322	*p* = 0.001 *
SOD	*p* = 0.488	*p* = 0.002 *
CAT	*p* = 0.566	*p* = 0.623

**Table 3:** Morphometrical Placental characteristics of mothers regarding the presence of COVID-19. Results are presented as the frequency of the observed entity in percentage (%).

Placental Characteristics	Thrombosis	Avascular Villi	Deposits of Fibrines	Villous Stromal Vascular Karyorrhexis	Obliteration of Blood Vessels	Vascular Ectasia	Delayed Villous Maturation	Placental Infarction	Retroplacental Hemorrhage	Hypoplasia of Distal Villus	Accelerated Villous Maturation	Decidual Arteriopathy	Inflammatory Changes
COVID-19 participants	+ [6/28] [21.4%]	++ [9/28] [32.1%]	+ [1/28] [3.57%]	[0/28] [0%]	+++ [12/28] [42.8%]	++ [8/28] [28.5%]	[0/28] [0%]	+ [3/28] [10.7%]	++ [7/28] [25%]	+ [2/28 [7.14%]	++ [8/28] [28.5%]	[0/28] [0%]	+ [4/28] [14.3%]
Non-COVID-19 participants	+ [1/22] [4.54%]	+ [1/22] [4.54%]	[0/22] [0%]	[0/22] [0%]	+ [1/22] [4.54%]	- [0/22] [0%]	+ [1/22] [4.54%]	[0/22] [0%]	- [0/22] [0%]	[0/22] [0%]	++ [4/22] [18.1%]	[0/22] [0%]	[0/22] [0%]

+ = presence of entity in 10%. ++ = presence of entity in 25%. +++ = presence of an entity in 40% and more.

## Data Availability

All data is available at the request of the corresponding author.

## Abbreviations

The following abbreviations are used in this manuscript:

COVID-19	Coronavirus disease 2019
NICU	Neonatal Intensive care unit
SARS-CoV-2	Severe Acute Respiratory Syndrome Coronavirus 2
FVM	Fetal Vascular Malperfusion
MVM	Maternal Vascular Malperfusion
VTR	Venous Thrombosis
ROS	Reactive Oxygen Species
RNS	Reactive Nitric Species
CRS	Chlorine Reactive Species
TAS	Total Antioxidative Status
CAT	Catalase
GPX	Glutathione peroxidase
SOD	Superoxide dismutase
IUGR	Intrauterine Grow Restriction
TBARS	Thiobarbituric acid Reactive Substances
GSH	Glutathione
RT-PCR	Reverse Transcription Polymerase Chain Reaction
